# Predictors of nutritional health risks among midwives in the context of midwifery work

**DOI:** 10.3389/fpubh.2025.1749254

**Published:** 2026-01-14

**Authors:** Aleksandra Łopatkiewicz, Olga Barbarska, Iwona Kiersnowska, Gabriel Pesta, Beata Guzak, Lucyna Kwiećkowska, Edyta Krzych-Fałta

**Affiliations:** 1 Department of Nursing Propaedeutics, Faculty of Health Sciences, Medical University of Warsaw, Warsaw, Poland; 2 School of Medical and Health Sciences, VIZJA University, Warsaw, Poland; 3 StatMed – Student Science Clubs, Department of Nursing Propaedeutics, Faculty of Health Sciences, Medical University of Warsaw, Warsaw, Poland; 4 Department of Lifestyle Medicine, School of Public Health, Centre of Postgraduate Medical Education, Warsaw, Poland

**Keywords:** BMI, C&RT, EAT-26, eating disorders, midwives, QWI, workload

## Abstract

**Background:**

Specific occupational factors among midwives—such as shift work, night duties, and significant professional responsibilities—are likely to contribute to a high perceived workload within this group. These factors may increase the risk of nutrition-related health issues, including abnormal body weight and disordered eating behaviors. The study aimed to assess the prevalence and key occupational predictors of such risks in this population.

**Methods:**

A cross-sectional preliminary study was conducted among 703 midwives in Poland. Disordered eating behaviors were evaluated using the Eating Attitudes Test-26 (EAT-26), while workload intensity was measured with the Quantitative Workload Inventory (QWI). Data were analyzed using non-parametric statistical tests and a Classification and Regression Tree (C&RT) model with V-fold cross-validation to identify the most influential predictors of nutritional health risk.

**Results:**

A Classification and Regression Tree (C&RT) model was used to identify key predictors of nutritional health risk, defined as meeting at least one of the following criteria: abnormal BMI, EAT-26 score > 20, behavioral indicators of disordered eating, or a history of treatment for an eating disorder. Of the participants, 56.76% (*n* = 399) were classified as being at nutritional health risk. The most salient predictors included work experience (more than 17.5 years), duration of night shift work, and QWI score.

**Conclusions:**

Night shift work alone was not a significant factor in the model. Work experience and workload intensity are key predictors of nutrition-related health risks among midwives. These findings highlight the need for workplace-level policies that address long-term workload burden—such as schedule optimization, limits on prolonged night-shift exposure, and improved access to psychological and nutritional support. Future research should further examine modifiable organizational factors and evaluate targeted interventions aimed at reducing nutrition-related risks in midwifery settings.

## 1 Introduction

The provision of midwifery care is demanding and frequently associated with emotional exhaustion resulting from work overload (1). Contemporary midwifery is grounded in a philosophy that places women and their families at the center of care while emphasizing ethical and evidence-based practice (2). A global shortage of midwives persists, with many professionals leaving the field primarily due to challenging working conditions, including excessive workload and inadequate professional support (3, 4). The Work, Health and Emotional Lives of Midwives (WHELM) program, which assessed midwives' well-being across twelve countries, revealed that midwives often feel overwhelmed, lack autonomy, experience a sense of underappreciation, and receive insufficient support (2). Together, these findings illustrate that occupational stress is deeply embedded in midwifery practice and may contribute to a range of adverse health consequences. Rather than reflecting isolated concerns, high job demands and chronic workload pressures create cumulative strain, which in turn diminishes professional confidence and is a significant contributor to dissatisfaction with midwifery as a career (5). Importantly, this chronic strain has been linked to lifestyle disruptions, including irregular dietary patterns and increased metabolic health risks (5–7).

In Poland, midwives play an essential role in the healthcare system, not only in maternal care but also in a wide range of preventive and health education initiatives. Unlike in many European countries where midwifery is a specialization pursued after nursing, Poland recognizes midwifery as an independent profession with its own educational pathway. The current bachelor-level training prepares midwives for broad clinical responsibilities across perinatal, neonatal, and gynecologic care (8, 9). These extensive responsibilities, combined with workforce shortages, contribute to high job demands and may intensify work-related stress.

An expanding body of evidence suggests that, due to high job demands and shift work, midwives may be particularly vulnerable to nutrition-related health risks, including abnormal body weight and disordered eating behaviors (10, 11). However, despite these indications, empirical research focusing specifically on midwives remains extremely limited. Most available evidence concerns nurses or mixed samples, making it difficult to distinguish risk patterns unique to the midwifery profession. One of the few large-scale analyses including midwives showed that over 60% of nurses and midwives were outside the healthy weight range, with obesity rates exceeding population norms by 1.7%−3.7% across three countries (Australia, New Zealand, UK) (12). Such findings indirectly suggest elevated vulnerability in midwifery, yet dedicated studies examining nutritional health risks in this profession—particularly those integrating behavioral and occupational determinants—are scarce.

Nutritional health risks encompass a spectrum of issues, such as abnormal body weight, disordered eating behaviors, and inadequate dietary patterns, all of which may carry serious long-term health implications. Eating disorders (EDs) are complex psychiatric conditions marked by severe disruptions in eating behavior, body image dissatisfaction, and an intense preoccupation with weight and food. These disorders are formally recognized in the Diagnostic and Statistical Manual of Mental Disorders (DSM-5) and the International Classification of Diseases (ICD-11) as serious mental health conditions with potentially severe medical and psychological consequences (13, 14).

Importantly, nutritional health risks extend beyond clinically diagnosed EDs and include body weight abnormalities—both underweight and overweight/obesity—which are associated with increased morbidity and mortality (15, 16). Abnormal BMI levels have been linked to higher risks of metabolic disorders, cardiovascular complications, and reduced quality of life. Certain EDs, such as anorexia nervosa and binge eating disorder, are closely associated with extreme BMI values—either significantly low or high—further intensifying health risks among affected individuals (17, 18).

While EDs have been widely studied in the general population, research on broader nutritional health risks among healthcare professionals—especially midwives—remains scarce. Nevertheless, existing evidence indicates that even during the early stages of their training, medical and healthcare students display elevated rates of ED symptoms compared to the general population. The pressures of academic achievement, demanding workloads, and high expectations in medical education may serve as early contributors to the development of disordered eating behaviors (19, 20). As these stressors often persist and intensify over the course of a healthcare professional's career, the risk of nutrition-related health problems—such as disordered eating behaviors and abnormal body weight—may further escalate.

Moreover, there is increasing interest in the application of artificial intelligence (AI) in public health, particularly in examining the complex interplay of cultural, social, and environmental factors that influence individual and community health outcomes (21). The use of AI methods such as decision trees allows us to understand nutrition-related health risks dependent on work-related factors.

Given the well-established links between occupational stress, shift work, and lifestyle patterns with adverse health outcomes, it is imperative to investigate whether midwives—who face substantial workplace demands—are particularly susceptible to nutrition-related health risks. These risks include both disordered eating behaviors and abnormal body weight. The present study aims to assess the prevalence and identify key predictors of nutrition-related health risks among midwives, with a focus on occupational and lifestyle-related determinants. By expanding the analysis beyond clinically diagnosed eating disorders, this research seeks to provide a more comprehensive understanding of the factors shaping midwives' dietary health and overall well-being.

## 2 Materials and methods

### 2.1 Participants and study design

The study sample comprised professionally active Polish midwives recruited from the Center for Postgraduate Education of Nurses and Midwives. Data collection was conducted using a traditional paper-and-pencil format. Of the 738 returned questionnaires, 35 were excluded due to incomplete responses. Consequently, 703 questionnaires were included in the final analysis. Ethical approval for the study was granted by the Bioethics Committee of the Medical University of Warsaw (approval number: AKBE/104/2025).

### 2.2 Sampling and sample size

In 2024, there were 28,800 midwives registered with the Chamber of Midwives in Poland. The group is dominated by women, with men being in the minority (*n* = 65) (22). The sample size was determined assuming a 96% confidence level, a 4% maximum error, and a population proportion of 0.5. Based on the above, the sample size was calculated to be 644 midwives. Participants were recruited using a convenience sampling approach during the national specialization examination for midwives conducted at the Center of Postgraduate Medical Education. Our study covered a sample of actively working midwives from across the country.

### 2.3 Data collection instruments

The study was conducted using a structured questionnaire comprising three sections: a customized demographic module and two standardized measurement tools. All data collected in the study were self-reported by participants.

The first instrument was the Eating Attitudes Test-26 (EAT-26) questionnaire (23), a widely recognized tool for assessing the risk of eating disorders. Although not diagnostic, the EAT-26 facilitates the identification of individuals who may exhibit disordered eating patterns and require further evaluation. It contains 26 items rated on a four-point Likert scale (0–4), with item 24 reverse-scored. In addition to the core attitudinal items, the EAT-26 also includes two supplementary subscales assessing behavioral indicators of disordered eating and history of treatment for an eating disorder. The validated Polish adaptation of the EAT-26 was used in this study (24).

The second tool was the 5-item Quantitative Workload Inventory (QWI) (25), designed to measure the perceived quantity and intensity of work performed. Each item is scored on a five-point Likert scale (1–5), with the total score ranging from 5 to 25. The Polish adaptation developed by Baka and Bazińska was used (26).

For the purposes of this study, nutrition-related health risk was defined based on several interrelated factors, including disordered eating behaviors and abnormal body weight. The classification was based on four key components, which were subsequently incorporated into the Classification and Regression Tree (C&RT) model analysis:

- Abnormal body weight according to BMI classification −287 participants (40.83%).
- EAT-26 score > 20−42 participants (5.97%).
- Behavioral indicators of disordered eating (ABCDE section of the questionnaire) −253 participants (35.99%).
- History of treatment for an eating disorder – 54 participants (7.68%).

**Figure 1:**
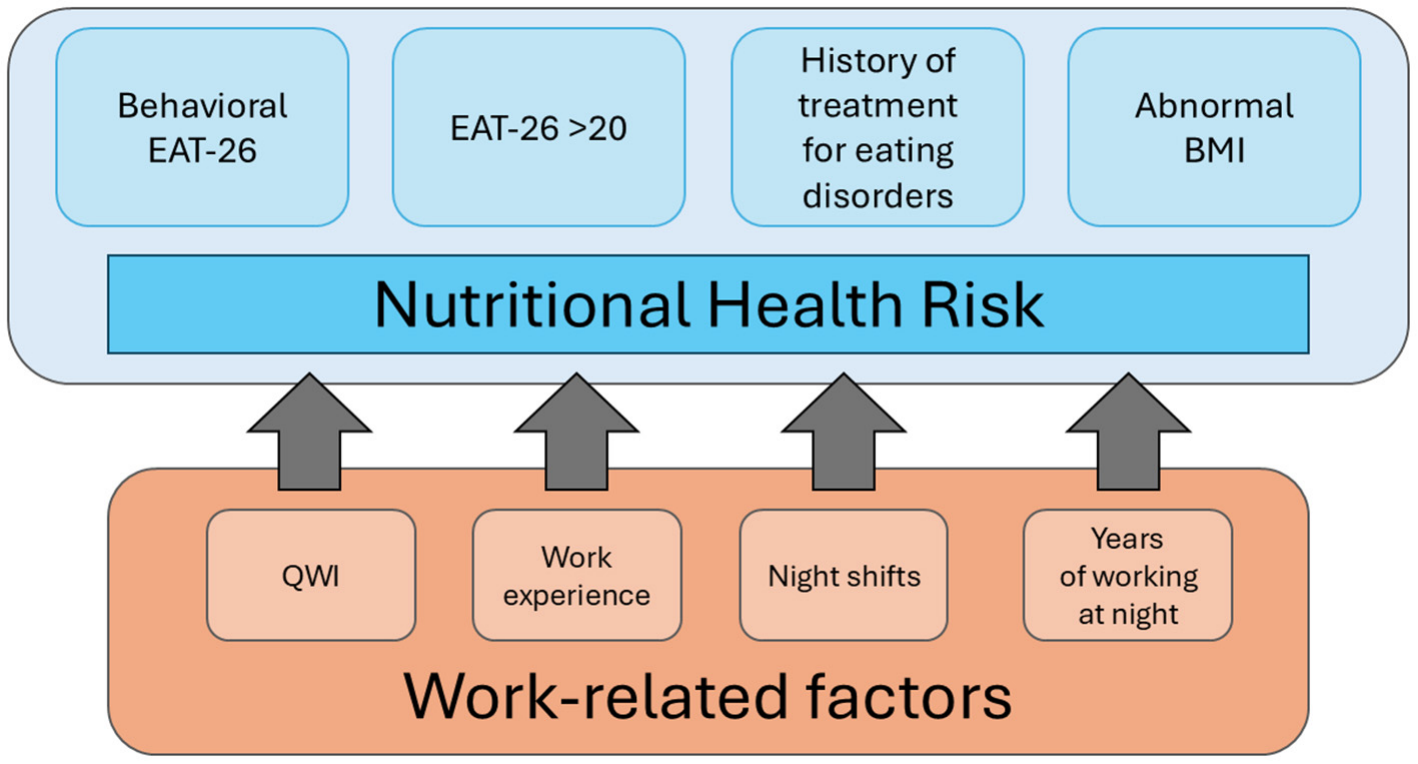
Graphical representation of components influencing health risks related to nutrition.

Participants who met at least one of these criteria were classified as being at risk for nutrition-related health issues (Figure 1). The definition of “nutrition-related health risk” adopted in this study is composite, encompassing both somatic and behavioral factors, consistent with the approach used in previous epidemiological analyses. The broad operationalization of risk is methodologically justified, as abnormal body weight, elevated scores on eating disorder screening tools, and history of ED treatment represent distinct, yet interrelated, dimensions of nutritional health. Population studies indicate that both abnormal BMI (27) and restrictive or compensatory behaviors identified using the EAT-26 (28) are independent predictors of health burden. In accordance with the EAT-26 framework, which refers to “extremely underweight” status but does not provide a numerical threshold for adults, we operationalized this category using the WHO criterion for severe thinness (BMI < 16 kg/m^2^). No participants in our sample met this threshold. Therefore, the “abnormal body weight” category (*n* = 287; 40.83%) consisted entirely of individuals with BMI ≥ 25 kg/m^2^ (overweight or obesity), while participants with BMI 16.0–18.49 kg/m^2^ were classified as underweight but not counted as “extremely underweight” for risk-classification purposes.

### 2.4 Statistical analysis

Group differences were analyzed using the Mann–Whitney *U* test and the chi-square test. Statistical significance was set at *p* < 0.05.

In this study, the Classification and Regression Trees (C&RT) method—specifically, the univariate split selection—was employed for statistical analysis. C&RT is a decision tree algorithm designed to detect patterns within data and to facilitate predictive modeling. The primary objective of the classification tree analysis and the use of single-factor segmentation was to achieve the most accurate possible prediction of the dependent variable (29).

A Classification and Regression Tree (C&RT) model was constructed using V-fold cross-validation to identify significant predictors of nutritional health risk The model criterion was a minimum number of offspring nodes equal to 20. The use of five-stage validation and minimum number of offspring nodes equal to 20 prevented overfitting. The dependent variable in the model was the presence of nutritional health risk. Independent variables included work-related factors such as length of professional experience, duration of night shift work, and workload as measured by the QWI score. The group predisposed to eating disorders constituted more than half of all surveyed midwives (399, 56.76%). Detailed results are presented in Tables 1, 2.

**Table 1:** Description of the dependent variable used to build the C&RT model (*n* = 399)*.

**Predictor (component)**	***n* (%)**
Abnormal body weight according to BMI classification	287 (40.83)
EAT-26 score > 20	42 (5,97)
Behavioral questions (ABCDE)	253 (35.99)
History of treatment for eating disorders	54 (7.68)

* The possibility of several predictors occurring concurrently.

**Table 2:** Differences between independent variables by group (*n* = 703).

**Independent variables**	**At nutritional health risk (*n* = 399)**	**No nutritional health risk (*n* = 304)**	***p***
Work experience	11.53 ± 10.16 6 (2–38)	8.54 ± 8.20 5 (2–42)	>0.001001*
Years of working at night	8.41 ± 9.14 4 (0–38)	6,69 ± 7.33 4 (0–35)	0.038001*
QWI	16.01 ± 5.20 15 (5–25)	15.83 ± 5.00 16 (5–25)	0.723
Night shifts	226 (74%, 34%)	279 (69.92%)	0.196

* *p* < 0.05.

The area under the curve (AUC) of the model was 0.64, and the Gini coefficient was 0.27. Reliability analysis confirmed high internal consistency of the applied questionnaires, with Cronbach's alpha coefficients of 0.87 for EAT-26 and 0.90 for QWI. All the calculations were performed using Statistica 13.3 software.

### 2.5 Characteristics of the study group

The study group consisted exclusively of women (*n* = 703; 100%). The mean age of participants was 34.00 ± 10.06 years, with a median age of 29 years (range: 24–65). The average length of work experience was 10.24 ± 9.47 years, with a median of 5 years (range: 2–42). The average number of years worked during night shifts was 7.67 ± 8.45 years, with a median of 4 years (range: 0–38). A detailed breakdown of participant characteristics is presented in Table 3.

**Table 3:** Characteristics of the study group.

**Catergory**	**Variable**	***n* (%)**
Gender	Female	703 (100%)
	Male	0
Marital status	Single	138 (19.63%)
	Married/in a relationship	535 (76.10%)
	Divorced	27 (3.84%)
	Widowed	3 (0.43%)
Education	Secondary/medical school	49 (6.97%)
	Bachelor's degree	117 (16.64%)
	Master's degree	533 (75.82%)
	Doctorate	4 (0.57%)
Work schedule	Night shifts	505 (71.83%)
	Morning shifts	122 (17.35%)
	12-h shifts (no nights)	76 (10.81%)

## 3 Results

The mean BMI of the participants was 24.71 ± 4.80, with a median of 23.80 (min: 16.30, max: 50.12). The lower quartile (Q1) was 21.36, and the upper quartile (Q3) was 26.89. A detailed breakdown of BMI classification based on WHO guidelines (30) is presented in Table 4.

**Table 4:** Distribution of participants by BMI classification (*n* = 703).

**Classification**	***n* (%)**
Underweight (16.0–18.4)	19 (2.7%)
Normal weight (18.5–24.9)	416 (59.17%)
Overweight (25.0–29.9)	169 (24.04%)
Obesity I (30.0–34.9)	76 (10.81%)
Obesity II (35.0–39.9)	14 (1.99%)
Severe obesity (≥40.0)	9 (1.28%)

A classification model was developed based on variables reflecting the specific nature of midwifery work. The final model comprised 10 split nodes and 11 terminal nodes. Among the predictors, the number of years working night shifts had the highest predictive rank (rank = 100). Other variables with substantial predictive value included total work experience (rank = 97) and the Quantitative Workload Inventory (QWI) score (rank = 45). The variable night shifts (rank = 12) was found to be insignificant in the model. Overall, the model indicates that work experience is the most influential predictor of nutrition-related health risk.

Among midwives with more than 17.5 years of professional experience (mean age = 51.13 ± 5.24 years; median = 51.5; range = 41–65), the strongest predictive factors were the number of years working night shifts (*n* = 101) and the QWI score, particularly for those with extensive night shift exposure (more than 15.5 years; *n* = 67).

In contrast, for midwives with 17.5 years or less of professional experience (*n* = 298; mean age = 29.52 ± 5.05 years; median = 28; range = 24–55), the QWI score emerged as the most significant predictor—especially in individuals with more than 3.75 years of total experience (*n* = 266), regardless of the duration of night shift work.

**Figure 2:**
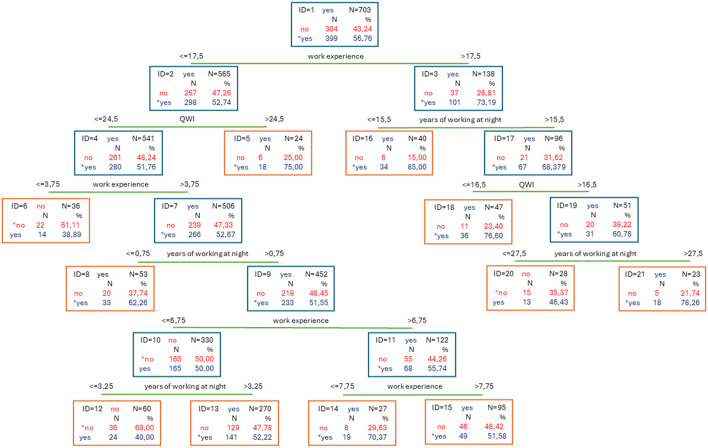
Model C&RT including factors related to the specifics of midwifery work.

A detailed breakdown of these patterns and their impact on nutritional health risk is provided in the subsequent section (Figure 2).

## 4 Discussion

The well-being of healthcare professionals is inextricably linked to patient safety outcomes. Poor emotional health among midwives is likely to negatively impact the quality, dignity, and safety of care provided to women and their newborns (31). Systematic reviews and meta-analyses based on large-scale observational studies have demonstrated associations between emotional well-being and working conditions in various healthcare professions—associations that are likely applicable to midwifery. Excessive work demands are commonly linked to burnout, particularly its core dimension of emotional exhaustion (32). The onset of depressive symptoms is often associated with high workload—characterized by elevated demands and limited autonomy—as well as a general lack of control over one's work environment (33). Additional occupational stressors such as effort–reward imbalance, low organizational justice, role-related pressure, insufficient support, interpersonal conflict, long working hours, and job insecurity are all strongly associated with elevated anxiety and stress, contributing to a persistent sense of overload (5, 34).

In the context of midwifery, specific sources of stress include shift work, high responsibility, excessive duties, time pressure, and fear of making irreversible mistakes, among others (35). Such work-related stress can overwhelm the interconnected biological, psychological, and social systems, ultimately leading to a range of negative somatic and psychological symptoms. Importantly, occupational stress influences not only job satisfaction but also health-related behaviors, including those linked to nutrition and eating habits (36). Given the significant impact of chronic work-related stress, it is crucial to consider its role in shaping lifestyle behaviors—particularly dietary patterns, physical activity, and self-care practices.

Despite growing awareness of mental health challenges among healthcare workers, nutrition-related health risks remain underdiagnosed and undertreated in this population. Several studies have identified a significant prevalence of overweight, obesity, and eating disorders among healthcare professionals—conditions that may co-occur and mutually reinforce one another (37, 38). Although validated screening instruments such as the Eating Attitudes Test-26 (EAT-26) (23) have been used to assess disordered eating, their application within medical settings remains limited. Research specifically examining the relationship between workload and nutrition-related health risks among healthcare workers is scarce; and in the case of midwives, virtually non-existent. Much of the existing literature focuses on physicians or general nursing staff, often neglecting the unique emotional and physical demands placed on midwives. Despite evidence highlighting their vulnerability to burnout and mental health challenges (39, 40), the association between occupational stressors and disordered eating in this group remains underexplored—underscoring the urgent need for further targeted research.

Although no studies have examined nutrition-related health risks specifically among midwives in other countries, several reports from broader healthcare worker populations offer partial points of reference. Research among nurses has shown that night shift work is associated with increased caloric intake, higher consumption of fats and sugars, greater meal frequency, and overall poorer diet quality (7, 11, 41, 42). These patterns align with evidence demonstrating that shift work substantially increases the risk of overweight and obesity, including a higher prevalence of abdominal obesity and greater risk among permanent night-shift employees (43). Although findings from nurses cannot be directly extrapolated to midwives due to differences in workload and professional responsibilities, they offer important contextual insight and highlight the broader relevance of occupational stressors and disrupted work schedules in shaping nutrition-related vulnerabilities.

In our study, we used one of the machine learning methods, namely the decision tree. Thanks to a method that combines classification and regression using supervised learning, classification trees are characterized by simplicity while maintaining strong predictive capabilities (44). To date, no studies have been conducted specifically on the prevalence of eating disorders among medical staff or the work-related factors that may contribute to their development. Consequently, this study conducted a preliminary analysis of factors influencing nutritional health risks among midwives resulting from the specific nature of their work.

### 4.1 BMI in the context of nutritional risk

Overweight and obesity are increasingly recognized as significant health concerns among nurses and midwives, with implications not only for their personal well-being but also for the quality of care they provide (12). According to the World Health Organization (WHO), a body mass index (BMI) between 25.0 and 29.9 kg/m^2^ is classified as overweight, while a BMI of 30.0 kg/m^2^ or higher is classified as obese (30). Excess body weight is associated with elevated risk for a range of chronic conditions, including cardiovascular disease, type 2 diabetes, hypertension, and certain cancers. Among healthcare professionals, these risks are compounded by demanding work schedules, shift duties, and elevated occupational stress—factors that can adversely affect dietary patterns and physical activity levels (45).

Although underweight (BMI < 18.5 kg/m^2^) also presents health risks—particularly with regard to nutritional deficiencies and compromised immune function—the increasing prevalence of overweight and obesity among midwives and nurses warrants particular attention. These conditions can have long-term consequences for physical health, mental well-being, and professional performance (41, 46).

In this study, abnormal BMI was among the predictors of eating disorder (ED) risk. Specifically, “extremely underweight” BMI was considered, based on guidance from the EAT-26 questionnaire (47). However, the EAT-26 guidelines do not clearly define “extremely underweight” for adults. Instead, they reference the NHANES III growth charts, which are based on pediatric percentiles developed for children and adolescents in the United States (48).

Given the absence of a standardized adult threshold in the EAT-26 framework—and considering that this study focused on a Polish adult population—we adopted the WHO classification for “severe thinness” (BMI < 16) as a proxy for “extremely underweight” (30). According to this criterion, no participants in our sample met the threshold for severe thinness. However, 19 individuals (2.7%) were classified as underweight (BMI < 18.5), placing them below the normal BMI range.

While low body weight is often emphasized in the context of eating disorder risk, it is equally important to acknowledge that excess body weight may also be associated with disordered eating behaviors. In our sample, 99 midwives (14.08%) were classified as obese, including 9 individuals (1.28%) with severe obesity. There is substantial evidence of co-occurrence between obesity and eating disorders, particularly binge eating disorder (BED). Research indicates that individuals with higher BMI are at increased risk of developing BED, and conversely, that BED significantly contributes to the development and persistence of obesity (46).

In our decision-tree model, BMI did not function as an isolated independent predictor of ED risk; however, abnormal BMI contributed to several classification pathways associated with elevated risk when combined with workload-related variables, such as long-term night-shift exposure or high perceived workload. This suggests that, in Polish midwives, BMI may interact with occupational stressors rather than exerting a direct effect on ED risk. Accordingly, the relationship observed in this study should be interpreted as preliminary and dependent on the broader occupational context, rather than as a standalone risk factor.

It is important to note that BMI is not a diagnostic criterion for eating disorders in either the Diagnostic and Statistical Manual of Mental Disorders (DSM-5) (13) or the International Classification of Diseases (ICD-11) (14). However, BMI may serve as a supporting indicator in clinical assessments. The EAT-26 questionnaire considers BMI only in the context of underweight, yet its threshold for “extremely underweight” remains undefined. If BMI is to function as a meaningful screening factor for nutritional health risks, its role requires further clarification and standardization.

### 4.2 Workload as a predictor of nutritional health risks

High workload has been consistently identified as a major factor adversely affecting emotional well-being in employees across high-demand professions. Although access to workplace resources—such as job autonomy and performance feedback—may help buffer some of these negative effects, such measures are frequently insufficient to fully mitigate the psychological burden (49). These insights underscore the critical importance of early intervention and continuous mental health monitoring for midwives, beginning at the onset of their careers.

All of these elements contribute to a distinct professional lifestyle among midwives—one that significantly influences eating behaviors, body image, and overall health. The physically and emotionally demanding nature of midwifery, coupled with extended work hours and unpredictable schedules, may disrupt regular eating patterns. These disruptions can lead to irregular meal timing and an increased risk of disordered eating behaviors (50).

High-stress occupations often prompt individuals to adopt maladaptive coping mechanisms, including unhealthy dietary behaviors and, in some cases, the development of eating disorders (51).

In the present study, although the total workload—as measured by the Quantitative Workload Inventory (QWI) score—did not emerge as an independent predictor, specific components such as professional experience and the cumulative duration of night shift work were identified as significant contributors to nutritional health risks.

Among midwives with more than 17.5 years of professional experience (mean age = 51.13 ± 5.24 years; median = 51.5; range = 41–65), the most salient predictive factors were years of working night shifts (*n* = 101) and the QWI score. These were especially influential among those with extensive night shift exposure (more than 15.5 years; *n* = 67). In this subgroup, a high QWI score appears to intensify the negative impact of long-term workload and night work on nutritional health risk.

In contrast, among midwives with 17.5 years or less of professional experience (*n* = 298; mean age = 29.52 ± 5.05 years; median = 28; range = 24–55), the QWI score alone emerged as the strongest predictor—particularly among individuals with more than 3.75 years of work experience (*n* = 266), regardless of the number of years worked during night shifts. This finding suggests that even in the early stages of a midwifery career, perceived workload is a critical determinant of stress-related health vulnerabilities.

### 4.3 Age and work experience

Our findings indicate that having more than 17.5 years of professional experience is the strongest predictor of nutrition-related health risks among midwives. Notably, midwives in this subgroup had a mean age of 51.13 ± 5.24 years (median = 51.5; range = 41–65), which closely aligns with the national average age of Polish midwives (51.4 years) (52). This correspondence suggests that a substantial portion of the national midwifery workforce may already fall into the highest-risk category, owing to both age and length of service.

Although the overall mean age in our study population was younger (34.0 ± 10.06 years), and the median work experience was only 5 years, the results clearly indicate that nutritional vulnerability increases with cumulative occupational exposure. Within the high-risk subgroup, two key factors—years worked during night shifts and overall QWI score—emerged as particularly strong predictors, especially among those with over 15.5 years of night shift experience.

Prolonged exposure to such conditions likely contributes to chronic occupational stress and emotional fatigue, which may promote maladaptive coping strategies such as irregular eating patterns, emotional eating, or increased reliance on calorie-dense, nutrient-poor convenience foods (51). These behaviors may be further intensified by age-related physiological changes, including a natural decline in metabolic rate and hormonal fluctuations during perimenopause and menopause, both of which are associated with increased weight gain and elevated BMI—particularly in the absence of regular physical activity and a balanced diet (53, 54).

### 4.4 Shift work

The adverse effects of shift work on both physical and mental health are well-documented. Research by Geiger-Brown and colleagues found that shift and night work significantly increase sleepiness at the end of shifts (55) and lead to disrupted sleep cycles (56). The specific impact of shift work may vary depending on whether the schedule is fixed or rotational. Permanent night shifts have been associated with a higher incidence of long-term sick leave when compared to exclusive day shifts (57), while rotating shifts have been linked to acute fatigue, increased error rates, and elevated alcohol consumption (6).

In our study, the second most influential predictor of nutritional health risk was the cumulative number of years spent working night shifts. This may be partially explained by evidence showing that nurses and midwives working night shifts tend to consume more total calories, fats, carbohydrates, and sugars than their daytime counterparts. This pattern was observed in a Polish study by Peplonska et al. (42), as well as in a large U.S. cohort study of nurses (NHS II) (58).

Furthermore, a study on a group of Lebanese nurses indicated that night shift work affects eating habits and food choices, leading to unhealthy dietary patterns (11). Night shift work has also been shown to influence dietary behaviors among healthcare professionals, leading to higher meal frequency and lower overall diet quality. Night shift workers tend to consume more snacks and fried foods high in saturated fats while consuming fewer fruits and vegetables compared to their day-shift counterparts (7, 41). These individuals also tend to extend their daily eating window, leading to increased caloric intake over a 24-h period (59). Such disruptions in eating behavior can elevate the risk of metabolic disorders and other health complications.

Moreover, a meta-analysis by Sun et al. (43) further confirmed the association between night shift work and increased risk of overweight and obesity. Similarly, findings from Lowden et al. demonstrated that shift workers are more susceptible than daytime workers to a wide range of health conditions, including obesity, cardiovascular disease, gastrointestinal problems, impaired glycemic control, and metabolic syndrome. While these conditions are linked in part to irregular diet and meal timing, other contributing factors include psychosocial stress, disrupted circadian rhythms, chronic sleep deprivation, insufficient physical activity, and limited recovery time (60).

### 4.5 Future directions

Future studies should explore additional factors such as lifestyle, eating habits, coping mechanisms, and social support to gain a more comprehensive understanding of the mechanisms contributing to nutrition-related health risks among midwives. Longitudinal studies could further clarify how prolonged exposure to occupational stress impacts eating behaviors over time and assess the effectiveness of workplace interventions aimed at reducing nutrition-related risks. By addressing these gaps, future investigations may contribute to the development of evidence-based strategies that enhance both the personal well-being of midwives and the quality of care provided to women and newborns.

## 5 Limitations

This study focused exclusively on variables related to workload intensity. Importantly, it did not incorporate direct measures of work-related psychological stress, which may also play a significant role in the development of disordered eating behaviors. The QWI captures participants' perceived workload but does not directly assess psychological stress. Therefore, while high workload may contribute to stress, the study did not include instruments measuring stress-related outcomes, such as burnout, anxiety, depression, or coping styles. This limitation should be considered when interpreting the associations between workload and nutrition-related health risk.

Despite the important contributions of this research, several limitations must be acknowledged. The reliance on self-reported instruments (EAT-26 and QWI) introduces the possibility of response bias, particularly given the sensitive nature of the questions. Moreover, the study's focus on a specific professional group—midwives—may limit the generalizability of the findings to other healthcare workers.

Another limitation concerns the interpretation of BMI as a risk factor for eating disorders. The EAT-26 questionnaire references BMI only in the context of underweight, without providing a standardized threshold for defining “extremely underweight” in adults. Additionally, the tool does not capture the complex bidirectional relationship between higher BMI and disordered eating behaviors, including conditions such as binge eating disorder.

## 6 Conclusions

Our findings underscore the significant influence of workload intensity and cumulative professional experience on the risk of eating disorders among midwives. The physically and emotionally demanding nature of the profession—coupled with irregular work schedules and elevated levels of occupational stress—appears to contribute meaningfully to the development of disordered eating behaviors. These results highlight the need to recognize nutrition-related vulnerabilities as an important component of occupational health in midwifery.

Given these challenges, targeted workplace interventions are warranted. Optimizing work schedules and shift rotations to reduce physical and psychological overload, implementing evidence-based stress-management programs, and providing access to psychological support services may help mitigate the risk of disordered eating. Nutritional education tailored to the realities of shift work, along with routine screening for disordered eating behaviors, could further strengthen preventive efforts. Creating a supportive, health-promoting work environment has the potential to improve both the well-being of midwives and the quality of care they provide.

## Data Availability

The datasets presented in this study can be found in online repositories. The names of the repository/repositories and accession number(s) can be found below: Zenodo repository: https://doi.org/10.5281/zenodo.16411450.
